# 

**Published:** 1854-11

**Authors:** 


					﻿No. VI.]
NOVEMBER.
[Vol. X.
BUFFALO
MEDICAL JOURNAL
AND
Jlbntbh) li cut civ
OT
MEDICAL AND SURGICAL SCIENCE.
AUSTIN FLINT, M. D.,	>
SANFORD B. HUNT, if D., J Editobs-
BUFFALO, N. Y.:
PUBLISHED BY THO JI AS <t LATHROPS.
Commercial Advertiser Buildings.
1854.
Price 82.50 per annum, in advance.
				

